# Awareness, Trial, and Current Use of Electronic Cigarettes in 10 Countries: Findings from the ITC Project

**DOI:** 10.3390/ijerph111111691

**Published:** 2014-11-13

**Authors:** Shannon Gravely, Geoffrey T. Fong, K. Michael Cummings, Mi Yan, Anne C. K. Quah, Ron Borland, Hua-Hie Yong, Sara C. Hitchman, Ann McNeill, David Hammond, James F. Thrasher, Marc C. Willemsen, Hong Gwan Seo, Yuan Jiang, Tania Cavalcante, Cristina Perez, Maizurah Omar, Karin Hummel

**Affiliations:** 1 Department of Psychology, University of Waterloo, 200 University Avenue West, Waterloo, Ontario, N2L 3G1, Canada; E-Mails: shannon.gravely@uwaterloo.ca (S.G.); m6yan@uwaterloo.ca (M.Y.); ackquah@uwaterloo.ca (A.C.K.Q.); 2 Ontario Institute for Cancer Research, MaRS Center, West Tower, 661 University Avenue, Suite 510, Toronto, Ontario, M5G 0A3, Canada; 3 Medical University of South Carolina, 171 Ashley Ave, Charleston, SC 29425, USA; E-Mail: cummingk@musc.edu; 4 The Cancer Council Victoria, 615 St. Kilda Road, Melbourne, Victoria 3004, Australia; E-Mails: Ron.Borland@cancervic.org.au (R.B.); Hua.Yong@cancervic.org.au (H.-H.Y.); 5 King’s College London, Strand, London, WC2R 2LS, UK; E-Mails: sara.hitchman@kcl.ac.uk (S.C.H.); ann.mcneill@kcl.ac.uk (A.M.); 6 School of Public Health and Health Systems, University of Waterloo, 200 University Avenue West, Waterloo, Ontario, N2L 3G1, Canada; E-Mail: dhammond@uwaterloo.ca; 7 Arnold School of Public Health, University of South Carolina, Colombia, SC 29208, USA; E-Mail: thrasher@mailbox.sc.edu; 8 Mexican Institute of Public Health, Universidad N. 655 Colonia Stana Maria Ahuacatitlan, Cerrada Los Pinos y Caminera C.P. 62100, Cuernavaca, Mor, Mexico; 9 Maastricht University (CAPHRI), P. Debyeplein 1, 6229 HA Maastricht, the Netherlands; E-Mails: marc.willemsen@maastrichtuniversity.nl (M.C.W.); Karin.hummel@maastrichtuniversity.nl (K.H.); 10 National Cancer Center of Korea, 323 Ilsan-ro, Ilsandong-gu, Goyang-si Gyeonggi-do, 410-769, Korea; E-Mail: hongwan@ncc.re.kr; 11 China Center for Disease Control and Prevention, 155 Changbai Road Changping District, Beijing 102206, China; E-Mail: jiangyuan88@vip.sina.com; 12 National Cancer Institute of Brazil, Praca Cruz Vermelha, 23, Centro, 20230-130, Rio de Janeiro, Brazil; E-Mails: taniac@inca.gov.br (T.C.); cperez@inca.gov.br (C.P.); 13 University Sains Malaysia, Jalan Sungai Dua, 11800 Georgetown, Pulau Pinang, Malaysia; E-Mail: maizurahomar@hotmail.com

**Keywords:** e-cigarette, electronic cigarette, nicotine, smoking, international

## Abstract

*Background*: In recent years, electronic cigarettes (e-cigarettes) have generated considerable interest and debate on the implications for tobacco control and public health. Although the rapid growth of e-cigarettes is global, at present, little is known about awareness and use. This paper presents self-reported awareness, trial and current use of e-cigarettes in 10 countries surveyed between 2009 and 2013; for six of these countries, we present the first data on e-cigarettes from probability samples of adult smokers. *Methods*: A cross-sectional analysis of probability samples of adult (≥ 18 years) current and former smokers participating in the International Tobacco Control (ITC) surveys from 10 countries. Surveys were administered either via phone, face-to-face interviews, or the web. Survey questions included sociodemographic and smoking-related variables, and questions about e-cigarette awareness, trial and current use. *Results*: There was considerable cross-country variation by year of data collection and for awareness of e-cigarettes (Netherlands (2013: 88%), Republic of Korea (2010: 79%), United States (2010: 73%), Australia (2013: 66%), Malaysia (2011: 62%), United Kingdom (2010: 54%), Canada (2010: 40%), Brazil (2013: 35%), Mexico (2012: 34%), and China (2009: 31%)), in self-reports of ever having tried e-cigarettes (Australia, (20%), Malaysia (19%), Netherlands (18%), United States (15%), Republic of Korea (11%), United Kingdom (10%), Mexico (4%), Canada (4%), Brazil (3%), and China (2%)), and in current use (Malaysia (14%), Republic of Korea (7%), Australia (7%), United States (6%), United Kingdom (4%), Netherlands (3%), Canada (1%), and China (0.05%)). *Conclusions*: The cross-country variability in awareness, trial, and current use of e-cigarettes is likely due to a confluence of country-specific market factors, tobacco control policies and regulations (e.g., the legal status of e-cigarettes and nicotine), and the survey timing along the trajectory of e-cigarette awareness and trial/use in each country. These ITC results constitute an important snapshot of an early stage of what appears to be a rapid progression of global e-cigarette use.

## 1. Introduction

The availability of less toxic forms of nicotine delivery products may represent a new paradigm for tobacco control by offering smokers an opportunity to obtain nicotine in ways that do not require inhaling tobacco smoke [1,2,3]. The apparent rapid ascent of alternative nicotine delivery systems (ANDS), particularly e-cigarettes, suggests that a significant number of cigarette smokers may be willing to try other forms of nicotine delivery [4,5]. However, there are many unanswered questions regarding the net public health benefits of e-cigarettes. For instance, concerns have been raised, among other things, about their effectiveness for smoking cessation, the extent of dual use that maintains cigarette addiction [5,6,7,8,9] and their facilitating smoking uptake among youth [6,10,11,12]. These and other issues have underscored the urgent need for research that would inform policies and regulations on e-cigarettes and other new and emerging nicotine delivery devices.

A recent systematic review by Pepper *et al.* presented a qualitative overview of the ANDS literature about awareness and use, and highlighted gaps in knowledge [5]. The authors make several suggestions for future research and in particular highlighted a need to better define the level of ANDS penetration to markets in different countries. For instance, considering the global popularity and ascendance of ANDS [4], evidence is very limited due to the fact that studies are generally limited in scope and design, thus making it difficult to make inferences about trial and use. In particular, study samples have been drawn from non-probability, self-selected samples [7,13,14,15,16,17,18,19,20], and nationally representative data are generally limited to the United States (US) [21,22,23,24,25,26,27] and Great Britain [28,29,30]. There are very few studies on ANDS (in particular, e-cigarettes) in other countries or across multiple countries, and of those, nearly all of them are from non-probability samples [31,32,33,34].

Only one study to date has presented an international comparison of e-cigarette awareness and use. Adkison *et al.* [35] presented data from Wave 8 (July 2010–June 2011) of the International Tobacco Control (ITC) Four Country Survey and examined levels of e-cigarette awareness and use in the US, United Kingdom (UK), Canada, and Australia. Data from 5939 adult current and former smokers across the four countries showed that 46.6% of the respondents were aware of e-cigarettes, 7.6% had tried them, and 2.9% were current users. Awareness was higher in the US (73%) and UK (54%) than in Canada (40%) and Australia (20%). Current use was also higher in the US (6%) and UK (4%) than in Canada (1%) and Australia (1%), showing that e-cigarettes are more common in countries where there are fewer or no restrictions.

The current study is the first of its kind to compare levels of e-cigarette awareness, trial, and current use among national representative samples from multiple countries with different economic levels and with tobacco control environments. The current study is an extension of Adkison *et al.*’s analysis of four high-income countries (US, UK, Canada, and Australia) [35]: the Australia data were updated (in 2013) and six other ITC countries were added (Brazil, China, Malaysia, Mexico, Netherlands, and the Republic of Korea). As of September 2014, the findings reported in this paper represent the first data on e-cigarettes from probability samples in five of these countries: Brazil, China, Malaysia, Mexico, and the Netherlands; and the first data from probability samples of adults in the Republic of Korea.

## 2. Methods 

### 2.1. Study Design and Procedure

A cross-sectional analysis was conducted from 10 countries participating in the International Tobacco Control (ITC) Surveys: The Netherlands, Republic of Korea, United States, Malaysia, United Kingdom, Canada, Brazil, Mexico, China, and Australia. The latest cohort dataset available in each country, conducted between 2009 and 2013, was included. Respondents were adult (≥age 18) current or former smokers. Current smokers were those who reported having smoked at least 100 cigarettes in their lifetime and who had smoked at least one cigarette in the past 30 days; former smokers were those who were smoking at the time of study initiation but had quit smoking at some point over the course of study follow-up. Recent quitters had stopped smoking in the last 6 months or less.

Methodological details for each country are available via the ITC Project website [36]. In brief, respondents for the ITC surveys were recruited through probability sampling of households in each country using random-digit dialing. Surveys were administered either via phone, face-to-face interviews, or the web. The individual initial response rates and individual average retention rates for subsequent waves for each country are presented in Table 1. Each survey wave used in the analysis includes data from both recontact (continuing) and replenishment (newly recruited) respondents, therefore the table shows a response rate for Wave 1 (except for Mexico, where the Wave 2 replenishment response rate is used as a surrogate) and an average of retention rates up to the wave being analyzed. The survey fieldwork or web survey was conducted in the country’s native language(s). The average length of the survey across all 10 countries was between 50–60 min for smokers and 30–40 min for former smokers. Ethics clearance was obtained from the Human Research Ethics Committee at the University of Waterloo and from other ethics committees in the other nine countries that required ethical clearance.

### 2.2. Measures

Questions about e-cigarettes included: (1) Have you ever heard of electronic cigarettes or e-cigarettes? (Responses: Yes or No); (2) Have you ever tried an e-cigarette? (Yes or No); and (3) Do you currently use e-cigarettes? The latter question about current use was not consistent between all countries. All responses were however dichotomized. Specifically, the question and responses for the Republic of Korea, US, UK, Canada, Australia, and Malaysia included: How often, if at all, do you currently use an electronic cigarette? (Responses: Daily, Less than daily, but at least once a week/Less than weekly, but at least once a month/ Less than monthly *versus* Not at all), The Netherlands: How often do you currently use an electronic cigarette? (Responses: Daily/Less than daily, but at least once a week/Less than weekly, but at least once a month /Less than monthly *versus* or have you stopped altogether?), and China: Are you currently using an electronic cigarette at least weekly? (Responses: Yes or No). For the countries that had multiple choices (all except for China) for current use, the variable was dichotomized into ‘yes’ or ‘no’ responses, where ‘yes’ corresponds to any form of use (daily, weekly or monthly) and ‘no’ corresponds to not being a current consumer (stopped or never used it).

## 3. Statistical Analysis

Descriptive statistics were computed on unweighted data to describe the characteristics of respondents (e.g., sex and age). All other results were based on analyses on weighted data to allow for prevalence estimates. The weights of each country were constructed individually by means of its sampling plan. For example, if simple random sampling was used, then the weights were inflated step by step to the household level then to the city/province level, then finally to the country representative level. If two-stage stratified sampling was used, then the weights were first inflated to the household level then to the cluster level, then to the strata level. These weights may not be country representative, so all inflated weights were then rescaled to the country sample size. The rescaled weights for each country were pooled in the analytic model to allow for cross-country comparisons. For details of the weight construction process for each country, please refer to the ITC Technical Reports [37]. 

Rates of e-cigarette awareness, trial, and current use were computed for smokers and former smokers. These rates were further broken down to present the proportion of current smokers and recent quitters (those that had quit smoking in the last 6 months) that were aware of, tried or currently used e-cigarettes. The analyses were conducted with SAS 9.2. Participant characteristics and survey details are presented in Table 1.

## 4. Results

Table 1 presents self-reported awareness, trial and current use of e-cigarettes by country. Awareness of e-cigarettes ranged from 88% in the Netherlands to 34% in China. Trial of e-cigarettes ranged from 20% in the Australia to 2% in China. Current use ranged from 14% in Malaysia to 0.05% in China.

## 5. Discussion 

This study of 10 countries provides a snapshot of the early stage of what appears to be a rapid progression in the global use of alternative nicotine delivery systems, such as e-cigarettes. In 6 countries—Netherlands, Brazil, Mexico, China, Republic of Korea, and Malaysia—we report the first data on e-cigarettes from probability samples of adults. The findings demonstrate considerable cross-country variation in both awareness and use of e-cigarettes. Some of the variability in awareness, trial, and use of e-cigarettes appears to be related to a combination of between-country differences in when the surveys were conducted (e.g., more recent surveys report higher rates of awareness and use), the regulatory status of e-cigarettes (e.g., Canada had lower rates than the US due to laws that restrict marketing of e-cigarettes), and levels of enforcement (e.g., Malaysia had high rates despite laws restricting access to product due to poor enforcement).

Interestingly, some of the cross-country differences do not seem readily explained by standard categories of countries such as high- *vs.* low-/middle-income. The relatively high rates in Malaysia run contrary to notions that e-cigarettes would be a less viable product in non-high income countries.

**Table 1:** Sociodemographic and Smoking Characteristics and Patterns of E-Cigarette Use.

Country	N	Dates of Data Collection (Survey Mid-Point ^‡^)	Survey Mode	Response Rate (Average Retention Rate)	N (%) Female	Age (Mean ± SD)	% Aware of E-Cigarettes (95% CI)	% Ever-Tried E-Cigarettes (95% CI)	% Currently Using E-Cigarettes (95% CI)
**China**	5583	May 2009–Oct 2009 (Jul-2009)	Face-to-Face	52.8% ^**^ (81.0%)	297 (5%)	50 ± 13	31% (28.7–34.0)	2% (1.8, 2.9)	0% (0.0–0.1)
*Cigarette Smokers*	*5209*				*270 (5%)*	*50 ± 13*	*31%* (28.4–33.8)	*2%* (1.8–3.0)	*~ 0%*
*Recent quitters*	*103*				*7 (7%)*	*52 ± 13*	*40%* (29.9–50.8)	*2%* (0–4.4)	*0%*
**United Kingdom**	1325	Jul 2010–Jun 2011 (Aug-2010)	Web or Phone	37.8% (72.8%)	726 (55%)	49 ± 13	54% (50.9–57.9)	10% (7.1–12.1)	4% (2.5–6.5)
*Cigarette Smokers*	*977*				*544 (56%)*	*49 ± 13*	*56%* (51.9–60.0)	*11%* (8.0–13.9)	*5%* (2.8–7.0)
*Recent quitters*	*77*				*46 (60%)*	*47 ± 13*	*60%* (44.4–74.9)	*16%* (0–32.9)	*11%* (0–28.5)
**United States**	1520	Jul 2010–Jun 2011 (Aug-2010)	Web or Phone	25.6% (63.4%)	805 (53%)	51 ± 13	73% (70.5–76.4)	15% (12.1–17.8)	6% (3.6–7.6)
*Cigarette Smokers*	*1262*				*671 (53%)*	*52 ± 13*	*74%* (70.5–76.8)	*18%* (14.2–21.0)	*6%* (4.0–8.8)
*Recent quitters*	*63*				*28 (44%)*	*50 ± 14*	*72%* (54.6–90.0)	*10%* (1.0–19.9)	*7%* (0–15.8)
Canada *	1581	Jul 2010–Jun 2011 (Aug-2010)	Web or Phone	49.5% (73.0%)	872 (55%)	47 ± 12	40% (36.5–42.6)	4% (2.7–5.3)	1% (0.6–2.1)
*Cigarette Smokers*	*1243*				*683 (55%)*	*48 ± 12*	*40%* (36.1–43.0)	*4%* (2.9–6.0)	*2%* (0.8–2.7)
*Recent quitters*	*74*				*34 (46%)*	*47 ± 14*	*39%* (25.2–53.7)	*5%* (0–12.4)	*0%* -
**Republic of Korea**	1753	Oct 2010–Dec 2010 (Nov-2010)	Phone	14.5% (50.3%)	83 (5%)	49 ± 16	79% (77.0–81.2)	12% (10.4–14.1)	7% (5.4–8.4)
*Cigarette Smokers*	*1560*				*76 (5%)*	*49 ± 16*	*80%* (77.6–82.0)	*13%* (10.9–14.9)	*7%* (5.7–8.9)
*Recent quitters*	*51*				*2 (4%)*	*51 ± 15*	*75%* (61.0–89.3)	*23%* (9.3–36.5)	*13%* (1.5–24.1)
Malaysia *	1998	May 2011–Feb 2012 (May-2011)	Phone	N/A (56.7%)	22 (1%)	33 ± 23	62% (57.5–66.1)	19% (16.2–22.6)	14% (11.6–15.7)
*Cigarette Smokers*	*1773*				*16 (1%)*	*31 ± 12*	*62%* (57.4–67.0)	*21%* (17.3–24.2)	*15%* (12.4–17.0)
*Recent quitters*	*69*				*3 (4%)*	*32 ± 14*	*69%* (53.1–85.8)	*13%* (3.2–22.2)	*6%* (0.4–11.5)
Mexico *^,†^	2129	Oct 2012–Dec 2012 (Nov-2012)	Face-to-Face	80.7% (73.6%)	801 (38%)	41 ± 15	34% (30.0–37.5)	4% (3.1–5.8)	-
*Cigarette Smokers*	*1747*				*646 (37%)*	*40 ± 15*	*34%* (30.0–37.8)	*5%* (3.4–6.7)	*-*
*Recent quitters*	*70*				*25 (36%)*	*41 ± 15*	*48%* (28.5–68.0)	*3%* (0.02–6.9)	*-*
Brazil *^,†^	1181	Oct 2012–Feb 2013 (Jan-2013)	Phone	10.6% (41.4%)	767 (65%)	49 ± 14	35% (31.6–38.5)	4% (2.3–5.8)	-
*Cigarette Smokers*	*1059*				*695 (66%)*	*49 ± 13*	*35%* (31.1–38.5)	*4%* (2.4–6.3)	*-*
*Recent quitters*	*44*				*25 (57%)*	*46 ± 13*	*36%* (19.7–52.2)	*4%* (0–10.1)	*-*
Australia *	1492	Feb 2013–Sep 2013 (Mar-2013)	Web or Phone	45.8% (74.5%)	801 (54%)	47 ± 13	66% (62.7–69.1)	20% (17.1–22.9)	7% (4.7–8.5)
*Cigarette Smokers*	*1093*				*586 (54%)*	*48 ± 13*	*69%* (65.6–73.0)	*24%* (20.4–27.7)	*9%* (6.2–11.4)
*Recent quitters*	*88*				*46 (52%)*	*44 ± 13*	*55%* (41.2–68.1)	*16%* (6.8–26.0)	*2%* (0–5.4)
**Netherlands**	1849	May 2013–Jun 2013 (May-2013)	Web	64.6% (81.3%)	907 (49%)	40 ± 15	88% (86.4–90.4)	19% (16.4–20.7)	3% (2.4–4.1)
*Cigarette Smokers*	*1420*				*686 (48%)*	*41 ± 15*	*87%* (84.8–89.6)	*20%* (17.4–22.5)	*4%* (2.8–5.0)
*Recent quitters*	*284*				*154 (54%)*	*38 ± 15*	*92%* (88.9–95.5)	*14%* (8.9–19.5)	*1%* (0–1.9)

Notes: Between-country comparisons cannot be made due to differences in survey timing and sequence of questioning in the survey; *Smokers* and *Recent quitters* categories will not add to the overall total because ‘recent quitters’ have been restricted to those who reported quitting within 6 months of the survey; Response rate for each country are for Wave 1 (except for Mexico, where the Wave 2 replenishment response rate is used as a surrogate) and an average of retention rates up to the wave being analyzed are reported above. Sample characteristics (gender and age) are unweighted and all other results are weighted by the rescaled cross-sectional weights; Prevalence estimates were rounded to the nearest whole number; SD = Standard deviation; CI = Confidence interval; N/A = Not available; * Countries where the sale of e-cigarettes containing nicotine is banned or restricted; ** estimated (exact rate could not be obtained); ^†^ The question about current use of e-cigarettes was not asked in the survey; **^‡^** The **‘**survey date mid-point’ is the month/year on which 50% of the respondents had successfully completed the survey for that wave. Dates are listed in chronological order (earliest to latest date surveyed).

It may well be the case that although some of the between-country differences may be at least partially explained by legal restrictions, as discussed above, that the variability between countries may be due to conditions specific to those countries such as the presence or absence of entrepreneurs and companies to bring a product that was until very recently, almost unknown. The uncertainty in explaining these differences across countries points to the need for case studies of the reasons for the ascendency of e-cigarettes in some countries and the relative absence of such products in others. In China, there was a very low rate of e-cigarette use. Although this may be surprising given that e-cigarettes were invented in China in 2003, and more than 90% of the e-cigarettes worldwide are produced in China, it should also be noted that China is the home of the world’s largest cigarette company, China National Tobacco Company (CNTC), which along with the State Tobacco Monopoly Administration, would have a strong interest in not supporting the emergence of e-cigarettes in China—a non-CNTC product. However, the atmosphere may be changing for e-cigarettes in China. A recent news report states that the CNTC is in the process of launching its own line of e-cigarettes [38].

These data confirm and extend previous knowledge of awareness, trial, and use in high-income countries (HICs). In particular they are consistent with data from large national surveys [21,23,28] providing further evidence of how levels of ANDS awareness and use have increased markedly in recent years; for example, the current study and published ITC Australian [35] data have shown that awareness of e-cigarettes increased from 20% in 2010 to 66% in 2013 and self-reported use from 1% in 2010 to 7% in 2013. Similarly, recent data from the Netherlands in 2013 demonstrate a rate of awareness (88%) that is higher than previously reported in the literature. One of the reasons for this is likely due to extensive advertising and promotion at some point during the life of the products, particularly in recent years. The dramatic rise in rates of ANDS awareness in countries where domestic advertising and product sale is banned or restricted is due to many factors such as poor enforcement of laws surrounding ANDS, availability via the internet and the black market, and the legal sale of non-nicotine e-cigarettes. More in depth reasons for changes in awareness and use requires more extensive population-based studies that will examine the changing political, cultural, regulatory and economic landscapes of both tobacco and ANDS products. At this point in time it is difficult to keep up with ANDS examinations as regulations are constantly changing and the information about product safety and efficacy is always evolving. Future studies that examine these issues will be critical to informing debates on the regulatory strategies that are most likely to benefit public health. Moreover, more research is needed among the low- and middle-income countries (LMICs). The data reported herein suggest that rates of e-cigarette awareness, trial, and use in LMICs are catching up to rates in HICs; however, restrictions on the sale and marketing of ANDS, in Mexico and Brazil, for example, are likely to slow down the trajectory. The long-term success in both HICs and LMICs will depend on whether ANDS will be embraced as a smoking cessation tool and/or permitted for sale and if advertising is allowable. The continued rise in popularity will depend on many factors, thus what appears to be a sharp rise in popularity and use of ANDS, the future trajectory is currently unknown.

There are several limitations in this study. First, the timing of the surveys differed across countries, and so it is, as discussed above, difficult to make confident judgments about the reasons for differences across countries. Thus, the lower rates of e-cigarette awareness and use reported in some countries may be an artifact of when the survey was conducted since e-cigarette use has increased with time in all countries where there exist longitudinal data. Additionally, recent unpublished ITC data from Australia, Canada, UK, and US reveal a rapid increase in reported awareness and use of e-cigarettes between 2010 and 2013 [39]. Another limitation of this study is that the measures of e-cigarette use do not distinguish between those who may have only tried an e-cigarette once and those who use them regularly. There is some evidence to suggest that the rapid increase in the use of e-cigarettes is dominated by short-term trialing done in an effort to stop smoking rather than long-term use [17,18,19]. The current cross-sectional design does not allow us to disentangle usage patterns, which are likely to vary within and between countries. Longitudinal studies of ANDS use are necessary to examine the dynamic relation between smoked products and ANDS. Such studies would be particularly valuable to inform evidence-based approaches to regulation of e-cigarettes and other ANDS. Indeed, such longitudinal studies will be forthcoming from the ITC Project. Moreover, alternative nicotine delivery devices are rapidly changing and many newer products are entering the market. The ITC data reported here specifically define awareness, trial and use of e-cigarettes only. Thus the rates of current use may have been underestimated if previous e-cigarette users switched to another ANDS (such as a vape pen or e-hookah). Consideration to include other ANDS in survey questioning is important to reduce measurement bias and get a better idea of the use of ANDS in order to avoid underestimation of trial and usage prevalence rates. Finally, for some estimated percentages, particularly for the quitter group, the standard error is quite large, and thus the estimates should be interpreted with caution.

## 6. Conclusions

In summary, this report provides a brief overview of e-cigarette awareness and use across 10 different countries with diverse economies and tobacco control histories. Additional research is needed to understand the patterns of global ANDS use in greater detail, particularly on impact of e-cigarettes on dual use, quitting, and relapse among smokers, and initiation of smoking among youth. There is a need for longitudinal studies to focus on the impact of ANDS, and address the critical question whether e-cigarettes represent a positive, negative, or mixed phenomenon for tobacco control and for public health. This will highly depend on studies that are able to measure and understand the interplay between ANDS and combustible tobacco products. The impact of ANDS marketing on the uptake of smoking by young people, along with the impact of e-cigarettes on smokers is necessary to make evidence-based judgments about the net burden of ANDS on the tobacco pandemic, within and across countries.
