# Correction: Gravely, S., *et al.* Awareness, Trial, and Current Use of Electronic Cigarettes in 10 Countries: Findings from the ITC Project. *Int. J. Environ. Res. Public Health* 2014*, 11,* 11691–11704

**DOI:** 10.3390/ijerph120504631

**Published:** 2015-04-27

**Authors:** Shannon Gravely, Geoffrey T. Fong, K. Michael Cummings, Mi Yan, Anne C. K. Quah, Ron Borland, Hua-Hie Yong, Sara C. Hitchman, Ann McNeill, David Hammond, James F. Thrasher, Marc C. Willemsen, Hong Gwan Seo, Yuan Jiang, Tania Cavalcante, Cristina Perez, Maizurah Omar, Karin Hummel

**Affiliations:** 1 Department of Psychology, University of Waterloo, 200 University Avenue West, Waterloo, Ontario, N2L 3G1, Canada; E-Mails: shannon.gravely@uwaterloo.ca (S.G.); m6yan@uwaterloo.ca (M.Y.); ackquah@uwaterloo.ca (A.C.K.Q.); 2 Ontario Institute for Cancer Research, MaRS Center, West Tower, 661 University Avenue, Suite 510, Toronto, Ontario, M5G 0A3, Canada; 3 Medical University of South Carolina, 171 Ashley Ave, Charleston, SC 29425, USA; E-Mail: cummingk@musc.edu; 4 The Cancer Council Victoria, 615 St. Kilda Road, Melbourne, Victoria 3004, Australia; E-Mails: Ron.Borland@cancervic.org.au (R.B.); Hua.Yong@cancervic.org.au (H.-H.Y.); 5 King’s College London, Strand, London, WC2R 2LS, UK; E-Mails: sara.hitchman@kcl.ac.uk (S.C.H.); ann.mcneill@kcl.ac.uk (A.M.); 6 School of Public Health and Health Systems, University of Waterloo, 200 University Avenue West, Waterloo, Ontario, N2L 3G1, Canada; E-Mail: dhammond@uwaterloo.ca; 7 Department of Health Promotion, Education & Behavior, Arnold School of Public Health, University of South Carolina, Colombia, SC 29208, USA; E-Mail: thrasher@mailbox.sc.edu; 8 National Institute of Public Health, Universidad N. 655, Colonia Stana Maria Ahuacatitlan, C.P. 62100, Cuernavaca, Mexico; 9 Maastricht University (CAPHRI), P. Debyeplein 1, 6229 HA Maastricht, the Netherlands; E-Mails: marc.willemsen@maastrichtuniversity.nl (M.C.W.); Karin.hummel@maastrichtuniversity.nl (K.H.); 10 National Cancer Center of Korea, 323 Ilsan-ro, Ilsandong-gu, Goyang-si Gyeonggi-do, 410-769, Korea; E-Mail: hongwan@ncc.re.kr; 11 China Center for Disease Control and Prevention, 155 Changbai Road, Changping District, Beijing 102206, China; E-Mail: jiangyuan88@vip.sina.com; 12 National Cancer Institute of Brazil, Praca Cruz Vermelha, 23, Centro, 20230-130, Rio de Janeiro, Brazil; E-Mails: taniac@inca.gov.br (T.C.); cperez@inca.gov.br (C.P.); 13 University Sains Malaysia, Jalan Sungai Dua, 11800 Georgetown, Pulau Pinang, Malaysia; E-Mail: maizurahomar@hotmail.com

The authors wish to make the following amendments to their paper published in *International Journal of Environmental Research and Public Health* [1]. These amendments only apply to the data from Brazil:

1. Page 11692, the abstract, line 15, the data collection for “Awareness of e-cigarettes” in Brazil should be 37%, and the data for “Trial of Electronic Cigarettes” in Brazil should be 8%. The correct sentence should be “There was considerable cross-country variation by year of data collection and for awareness of e-cigarettes (Netherlands (2013: 88%), Republic of Korea (2010: 79%), United States (2010: 73%), Australia (2013: 66%), Malaysia (2011: 62%), United Kingdom (2010: 54%), Canada (2010: 40%), Brazil (2013: 37%), Mexico (2012: 34%), and China (2009: 31%)), in self-reports of ever having tried e-cigarettes (Australia, (20%), Malaysia (19%), Netherlands (18%), United States (15%), Republic of Korea (11%), United Kingdom (10%), Brazil (8%), Mexico (4%), Canada (4%), and China (2%)), and in current use (Malaysia (14%), Republic of Korea (7%), Australia (7%), United States (6%), United Kingdom (4%), Netherlands (3%), Canada (1%), and China (0.05%))”.
2. Page 11696, Table 1, the data for Brazil are corrected. There are no changes in the data of any of the other countries. The corrected Table 1 is as follows:

**Table 1:** Sociodemographic and Smoking Characteristics and Patterns of E-Cigarette Use.

Country	N	Dates of Data Collection (Survey Mid-Point ^‡^)	Survey Mode	Response Rate (Average Retention Rate)	N (%) Female	Age (Mean ± SD)	% Aware of E-Cigarettes (95% CI)	% Ever-Tried E-Cigarettes (95% CI)	% Currently UsingE-Cigarettes (95% CI)
**China**	5583	May 2009–Oct 2009 (Jul-2009)	Face-to-Face	52.8% ^**^ (81.0%)	297 (5%)	50 ± 13	31% (28.7–34.0)	2% (1.8, 2.9)	0% (0.0–0.1)
*Cigarette Smokers*	*5209*				*270 (5%)*	*50 ± 13*	*31%* (28.4–33.8)	*2%* (1.8–3.0)	*~0%*
*Recent quitters*	*103*				*7 (7%)*	*52 ± 13*	*40%* (29.9–50.8)	*2%* (0–4.4)	*0%*
**United Kingdom**	1325	Jul 2010–Jun 2011 (Aug-2010)	Web or Phone	37.8% (72.8%)	726 (55%)	49 ± 13	54% (50.9–57.9)	10% (7.1–12.1)	4% (2.5–6.5)
*Cigarette Smokers*	*977*				*544 (56%)*	*49 ± 13*	*56%* (51.9–60.0)	*11%* (8.0–13.9)	*5%* (2.8–7.0)
*Recent quitters*	*77*				*46 (60%)*	*47 ± 13*	*60%* (44.4–74.9)	*16%* (0–32.9)	*11%* (0–28.5)
**United States**	1520	Jul 2010–Jun 2011 (Aug-2010)	Web or Phone	25.6% (63.4%)	805 (53%)	51 ± 13	73% (70.5–76.4)	15% (12.1–17.8)	6% (3.6–7.6)
*Cigarette Smokers*	*1262*				*671 (53%)*	*52 ± 13*	*74%* (70.5–76.8)	*18%* (14.2–21.0)	*6%* (4.0–8.8)
*Recent quitters*	*63*				*28 (44%)*	*50 ± 14*	*72%* (54.6–90.0)	*10%* (1.0–19.9)	*7%* (0–15.8)
Canada *	1581	Jul 2010–Jun 2011 (Aug-2010)	Web or Phone	49.5% (73.0%)	872 (55%)	47 ± 12	40% (36.5–42.6)	4% (2.7–5.3)	1% (0.6–2.1)
*Cigarette Smokers*	*1243*				*683 (55%)*	*48 ± 12*	*40%* (36.1–43.0)	*4%* (2.9–6.0)	*2%* (0.8–2.7)
*Recent quitters*	*74*				*34 (46%)*	*47 ± 14*	*39%* (25.2–53.7)	*5%* (0–12.4)	*0%* -
**Republic of Korea**	1753	Oct 2010–Dec 2010 (Nov-2010)	Phone	14.5% (50.3%)	83 (5%)	49 ± 16	79% (77.0–81.2)	12% (10.4–14.1)	7% (5.4–8.4)
*Cigarette Smokers*	*1560*				*76 (5%)*	*49 ± 16*	*80%* (77.6–82.0)	*13%* (10.9–14.9)	*7%* (5.7–8.9)
*Recent quitters*	*51*				*2 (4%)*	*51 ± 15*	*75%* (61.0–89.3)	*23%* (9.3–36.5)	*13%* (1.5–24.1)
Malaysia *	1998	May 2011–Feb 2012 (May-2011)	Phone	N/A (56.7%)	22 (1%)	33 ± 23	62% (57.5–66.1)	19% (16.2–22.6)	14% (11.6–15.7)
*Cigarette Smokers*	*1773*				*16 (1%)*	*31 ± 12*	*62%* (57.4–67.0)	*21%* (17.3–24.2)	*15%* (12.4–17.0)
*Recent quitters*	*69*				*3 (4%)*	*32 ± 14*	*69%* (53.1–85.8)	*13%* (3.2–22.2)	*6%* (0.4–11.5)
Mexico *^,†^	2129	Oct 2012–Dec 2012 (Nov-2012)	Face-to-Face	80.7% (73.6%)	801 (38%)	41 ± 15	34% (30.0–37.5)	4% (3.1–5.8)	-
*Cigarette Smokers*	*1747*				*646 (37%)*	*40 ± 15*	*34%* (30.0–37.8)	*5%* (3.4–6.7)	*-*
*Recent quitters*	*70*				*25 (36%)*	*41 ± 15*	*48%* (28.5–68.0)	*3%* (0.02–6.9)	*-*
Brazil *^,†^	1215	Oct 2012–Feb 2013 (Jan-2013)	Phone	10.6% (41.4%)	787 (65%)	49 ± 14	37% (34.0–41.0)	8% (5.7–10.1)	-
*Cigarette Smokers*	*1090*				*714 (65%)*	*49 ± 14*	*37%* (33.7–41.0)	*8%* (5.8–10.7)	*-*
*Recent quitters*	*45*				*25 (56%)*	*46 ± 13*	*38%* (23.7–55.4)	*8%* (2.4–22.9)	*-*
Australia *	1492	Feb 2013–Sep 2013 (Mar-2013)	Web or Phone	45.8% (74.5%)	801 (54%)	47 ± 13	66% (62.7–69.1)	20% (17.1–22.9)	7% (4.7–8.5)
*Cigarette Smokers*	*1093*				*586 (54%)*	*48 ± 13*	*69%* (65.6–73.0)	*24%* (20.4–27.7)	*9%* (6.2–11.4)
*Recent quitters*	*88*				*46 (52%)*	*44 ± 13*	*55%* (41.2–68.1)	*16%* (6.8–26.0)	*2%* (0–5.4)
**Netherlands**	1849	May 2013–Jun 2013 (May-2013)	Web	64.6% (81.3%)	907 (49%)	40 ± 15	88% (86.4–90.4)	19% (16.4–20.7)	3% (2.4–4.1)
*Cigarette Smokers*	*1420*				*686 (48%)*	*41 ± 15*	*87%* (84.8–89.6)	*20%* (17.4–22.5)	*4%* (2.8–5.0)
*Recent quitters*	*284*				*154 (54%)*	*38 ± 15*	*92%* (88.9–95.5)	*14%* (8.9–19.5)	*1%* (0–1.9)

Notes: Between-country comparisons cannot be made due to differences in survey timing and sequence of questioning in the survey; *Smokers* and *Recent quitters* categories will not add to the overall total because ‘recent quitters’ have been restricted to those who reported quitting within 6 months of the survey; Response rate for each country are for Wave 1 (except for Mexico, where the Wave 2 replenishment response rate is used as a surrogate) and an average of retention rates up to the wave being analyzed are reported above. Sample characteristics (gender and age) are unweighted and all other results are weighted by the rescaled cross-sectional weights; Prevalence estimates were rounded to the nearest whole number; SD = Standard deviation; CI = Confidence interval; N/A = Not available; * Countries where the sale of e-cigarettes containing nicotine is banned or restricted; ** estimated (exact rate could not be obtained); ^†^ The question about current use of e-cigarettes was not asked in the survey; **^‡^** The ‘survey date mid-point’ is the month/year on which 50% of the respondents had successfully completed the survey for that wave. Dates are listed in chronological order (earliest to latest date surveyed).

The authors would like to apologize for any inconvenience caused to readers by these changes.
